# Non-coding RNAs in breast cancer: with a focus on glucose metabolism reprogramming

**DOI:** 10.1007/s12672-023-00687-2

**Published:** 2023-05-19

**Authors:** Junjie Liang, Chun Ye, Kaiqin Chen, Zihan Gao, Fangguo Lu, Ke Wei

**Affiliations:** 1 Medical School, Hunan University of Chinese Medicine, Changsha, 410208 China; 2 Hunan Province Key Laboratory of Integrative Pathogen Biology, Hunan University of Chinese Medicine, Changsha, 410208 Hunan China

**Keywords:** miRNA, lncRNA, circRNA, Breast cancer cells, Glucose metabolism

## Abstract

Breast cancer is the tumor with the highest incidence in women worldwide. According to research, the poor prognosis of breast cancer is closely related to abnormal glucose metabolism in tumor cells. Changes in glucose metabolism in tumor cells are an important feature. When sufficient oxygen is available, cancer cells tend to undergo glycolysis rather than oxidative phosphorylation, which promotes rapid proliferation and invasion of tumor cells. As research deepens, targeting the glucose metabolism pathway of tumor cells is seen as a promising treatment. Non-coding RNAs (ncRNAs), a recent focus of research, are involved in the regulation of enzymes of glucose metabolism and related cancer signaling pathways in breast cancer cells. This article reviews the regulatory effect and mechanism of ncRNAs on glucose metabolism in breast cancer cells and provides new ideas for the treatment of breast cancer.

## Introduction

Breast cancer is one of the most common malignancies that threaten women's health. Its morbidity and mortality rates are among the highest among cancers in women. Although good progress has been made in the treatment of breast cancer in recent decades, the treatment system is imperfect, surgical and drug therapy is still not ideal, and the clinical prognosis is poor [1]. According to recent research, abnormal glucose metabolism in breast cancer cells is closely related to the poor prognosis of the disease. Compared with normal cells, glucose metabolism in cancer cells switches from oxidative phosphorylation (OXPHOS) to glycolysis and converts glucose to lactate, even when sufficient oxygen is present and mitochondrial function is intact. This phenomenon is also known as aerobic glycolysis or the "Warburg effect" [2]. This reprogramming of metabolic pathways is closely associated with the activation of proto-oncogenes, transcription factors and related signaling pathways. In addition, oncogenes and tumor suppressor genes can activate the expression of key glycolytic enzymes and glucose transporter proteins, thereby enhancing aerobic glycolysis in tumor cells. With the in-depth study of the mechanism of tumor cell metabolism, targeting the glucose metabolism of tumor cells is considered a promising way of cancer treatment, and the combination of chemotherapeutic agents and glycolysis inhibitors has emerged as a promising strategy to overcome anticancer drug resistance [3]. Non-coding RNAs (ncRNAs), a type of RNA lacking the function of a coding protein, have been closely associated with the reprogramming of glucose metabolism in cancer cells in recent years.

**Fig. 1:**
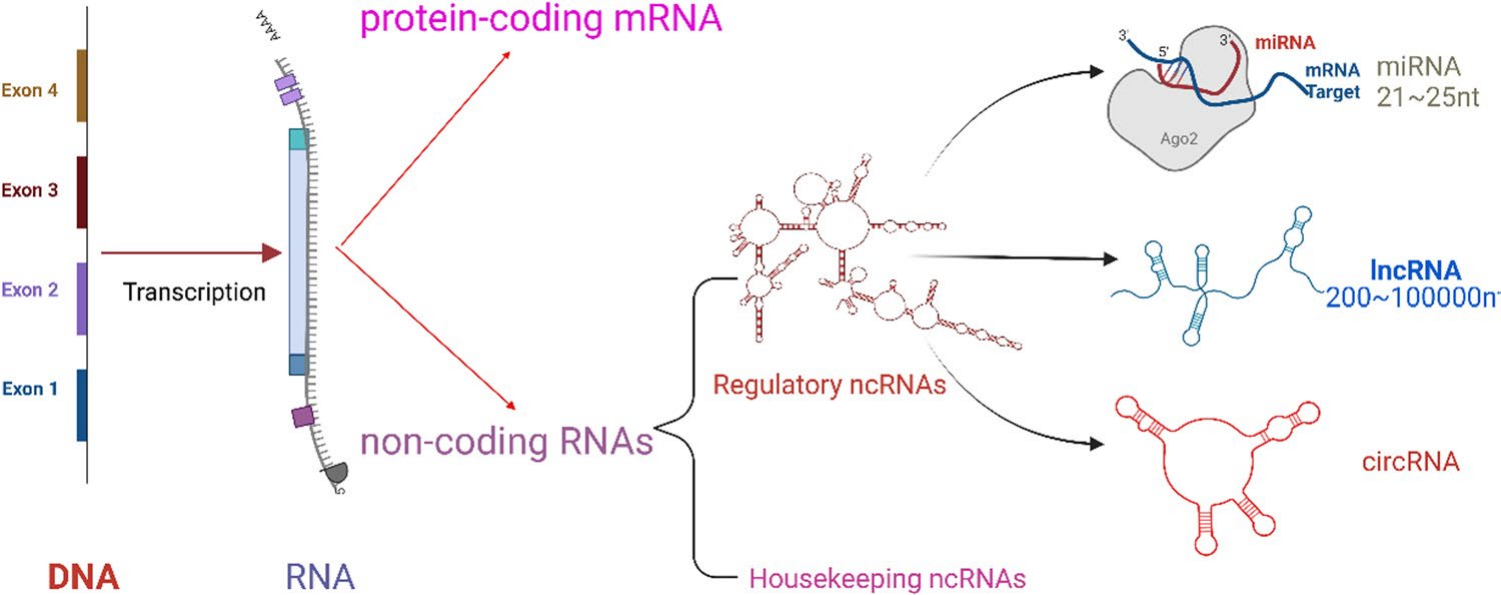
The generation and classification of non-coding RNA: In eukaryotes, about 90% of genes are transcribed genes, only about 2% of these transcribed genes encode proteins, and the remaining 98% do not have the function of encoding proteins, which are called non-coding RNAs. ncRNAs are mainly divided into housekeeping ncRNAs and regulatory ncRNAs according to their functions and regulatory ncRNAs are divided into miRNAs and lncRNAs according to their length

It is reported that only about 2% of genes in the human genome can encode proteins, while the remaining 98% are transcribed into ncRNA. Furthermore, ncRNAs can be divided into Housekeeping ncRNAs and Regulatory ncRNAs according to their length and function. Regulatory ncRNAs also include MicroRNA(miRNA), Long non-coding RNA(lncRNA), Circular RNA(circRNA), and so on [4] (Fig. 1 shows the generation and classification of non-coding RNA)*.* With the continuous improvement of ncRNA identification technology, researchers have gradually discovered the important role of ncRNAs in tumor development [5]. Recent studies have shown that ncRNAs can affect the gluconeogenic pathways of breast cancer by regulating enzymes, pathways and genes related to gluconeogenesis of breast cancer cells, which provides a new approach for the therapy of gluconeogenesis of breast cancer [6]. This review focuses on the regulatory role and related mechanisms of non-coding RNAs in the glucose metabolism pathway of breast cancer and summarizes and analyzes them to provide a reference for the search and development of new targets for the treatment of breast cancer.

## MiRNAs in breast cancer glucose metabolism

miRNA are small RNAs without protein-coding function that are widely expressed in eukaryotes. They are 21-25nt in length and can complement and bind to the 3′-untranslated region of the target mRNA, resulting in direct degradation or inhibition of translation of target genes, thus reducing their expression [7, 8]. In addition, miRNAs are usually involved in gene regulation at the post-transcriptional level. Previous studies have shown that miRNAs can be used as oncogenes to promote cancer cell invasion and metastasis. In contrast, some miRNAs can also act as tumor suppressor genes to prevent tumor development and extensively affect genomic regulation [9]. In recent years, miRNAs have been identified as important regulators of glucose metabolism in breast cancer cells and play important roles in tumor proliferation and metastasis [10]. As important targets of glucose metabolism in breast cancer, some miRNAs are of great research value in the occurrence and development of breast cancer.

### miRNA regulates glycolytic enzymes in breast cancer cells

#### HK2

Hexokinase (HK) is a rate-limiting enzyme that catalyzes the first step of glycolysis and can phosphorylate glucose into glucose-6-phosphate. Four HK isozymes have been found in mammals: HK1, HK2, HK3, and HK4 [11]. In breast cancer cells, overexpression of HK2 promotes tumor glycolysis and metastasis, leading to poor prognosis. HK2 is also an important target for cancer treatment. A growing number of studies have shown that targeting HK2 effectively controls glucose metabolism and influences cancer progression. MiR-155 is a type of miRNA mediated by inflammation and is closely related to miR-143. MiR-143 directly inhibits HK2 and negatively regulates glycolysis in breast cancer cells. miR-155 can inhibit the expression of miR-143 by targeting CCAAT enhancer binding protein β (C/EBPβ), resulting in upregulation of HK2 level and promotion of glucose metabolism in cancer cells [12]. miR-155 can also promote glycolysis in breast cancer through the PIK3R1-FOXO3A-cMYC pathway, miRNA-155 can antagonize FOXO3A, which can destroy the stability of c-MYC. In addition, c-MYC is the main regulatory factor for glycolysis in cancer cells. As a result, as FOXO3A levels decline, the glycolysis level of breast cancer cells increases, demonstrating that the miR-155/PIK3R1-FOXO3A-cMYC axis is critical in controlling glucose metabolism [13]. In addition, miR-216b is considered as a tumor suppressor that can directly downregulate HK2, block mTOR signaling pathway, and induce autophagy and apoptosis in cancer cells [14]. These results suggest that targeting downregulation of HK2 in breast cancer cells effectively inhibits the malignant grade of breast cancer. At the same time, it also provides an alternative treatment for breast cancer.

#### PKM2

In the last step of glycolysis, pyruvate kinase (PK) catalyzes the acidification of phosphoenolpyruvate (PEP) to pyruvate. PK can be divided into four subtypes: L, R, M1, and M2. The M2 subtype has received much attention in tumor research [15]. PKM2 plays an important role in the energy conversion of cells, which can switch the mode of energy supply from OXPHOS to aerobic glycolysis, increasing the lactic acid content [16]. In breast cancer cells, high expression of PKM2 accelerates malignant progression. Studies have shown that some miRNAs can target and regulate PKM2 expression. For example, miR-122 is highly secreted by tumor cells and promotes metastasis by adapting to the metabolic environment in the pre-metastatic niche, while down-regulation of PKM2 and glucose transporter type 1 (GLUT1) inhibits glucose metabolism in breast cancer cells [17]. Serguienko et al. discovered that miR-Let-7a inhibited MDA-MB-231 cell proliferation and specifically upregulated the levels of PMK2, OXPHOS, and reactive oxygen species (ROS) in triple-negative breast cancer (TNBC) and increased the sensitivity of breast cancer cells to the tumor suppressor doxorubicin in metastatic breast cancer [18]. In addition, Yao et al. found that miR-Let-7a-5p can regulate the Stat3/hnRNP-A1/PKM2 signaling pathway to inhibit aerobic glycolysis. They found that heterogeneous nuclear ribonucleoprotein (hnRNP)-A1 regulates the selective cleavage of PKM2 gene, thereby promoting the production of PKM2. Signal transducer and activator of transcription 3 (STAT3) can promote the transcription of (hnRNP)-A1, but miR-Let-7a-5p negatively regulates Stat3 and therefore decreases the level of PKM2 [19, 20]. In conclusion, PKM2, the key enzyme of aerobic glycolysis in cancer cells, plays an important role in malignant progression of breast cancer. Finding a breakthrough that targets PKM2 will be a promising direction for the treatment of breast cancer.

#### LDHA

Lactate dehydrogenase A (LDHA) can reduce pyruvate to lactic acid and catalyzes the final step of glycolysis. It is also a key enzyme in the regulation of aerobic glycolysis in cancer cells. The regulation of LDHA is considered to be an important way to affect the Warburg effect in tumors. miRNAs can be used as tumor suppressors to inhibit the expression of LDHA, weaken the Warburg effect in tumors, and inhibit the growth and metastasis of breast cancer. Some miRNAs such as miR-34a and miR-30a-5p negatively regulate LDHA levels and inhibit glycolysis in cancer cells by targeting LDHA, resulting in decreased glucose uptake and lactate production [21, 22]. With the advances in research, LDHA has emerged as a biomarker and therapeutic target for cancer, suggesting that targeting LDHA may be a reliable way to treat breast cancer [23].

#### Other related enzyme

**Fig. 2:**
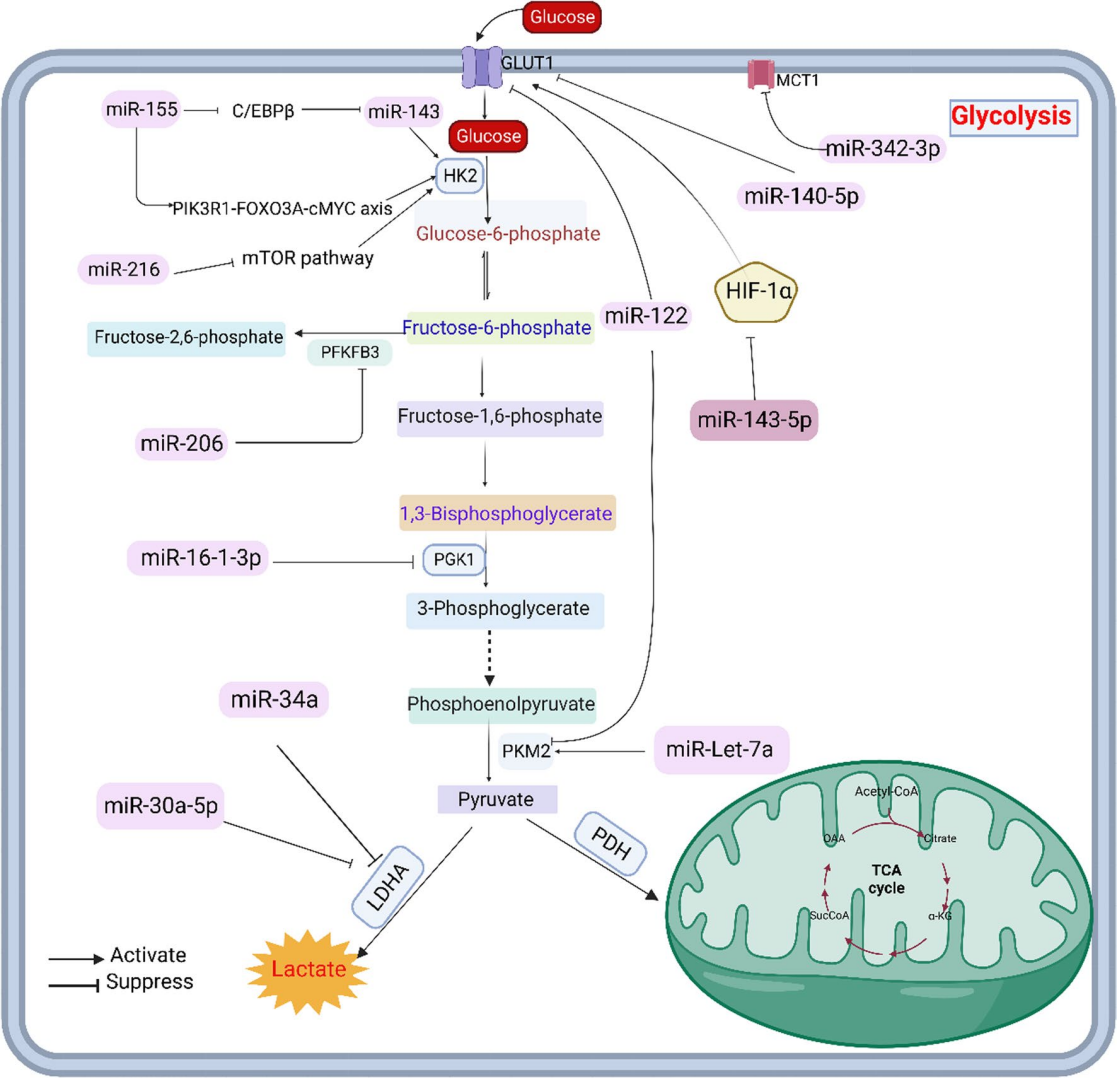
miRNAs can influence the process of glucose metabolism in breast cancer cells by regulating gluconeogenic enzymes. GLUT1, HK2, PGK1, PKM2, and LDHA can all be regulated by miRNAs

Phosphoglucosinase-like protein 5(PGM5) converts glucose-1-phosphate (G1P) to glucose-6-phosphate (G6P) and is upregulated in a variety of cancers, primarily as a biomarker to predict the prognosis of cancer patients. Studies have shown that low PGM5 expression is a sign of poor prognosis. It has been found that the high expression of PGM5 in Human breast cancer cell lines MCF7 and ZR75-1 can inhibit LDHA and slow down cancer progression, but miR-1224-3p can directly inhibit PGM5, which leads to the proliferation and metastasis of breast cancer cells [24]. Phosphoglycerate kinase 1 (PGK1) is the first glycolytic enzyme that produces adenosine triphosphate (ATP) in the aerobic glycolysis pathway, which is indispensable for the occurrence of the Warburg effect. By lowering PGK1 expression, miR-16-1-3p can decrease glucose absorption and lactate generation in MDA-MB-231 breast cancer cells, reducing the Warburg effect [25]. Fructose-6-phosphate-2-kinase/fructose-2-biphosphatase (PFK1 /FBPase1, PFKFB) is a bifunctional enzyme that effectively controls the rate of glycolysis in tumors (mainly divided into four isozymes, namely PFKFB1‒4) [26]. Related studies have shown that PFKFB3 is a direct target of miR-206, which inhibits glycolytic metabolism in tumor cells by downregulating PFKFB3 and acts as a tumor inhibitory factor. However, miR-206 is significantly downregulated in breast cancer, leading to an increase in PFKFB3 and further accelerating the progression of breast cancer[27] (Fig. 2 demonstrates the regulatory role of miRNA in breast cancer glycolysis).

### miRNA regulates glucose metabolic pathways and oncogenes in breast cancer cells

#### HIF-1α signaling pathway

Hypoxia-inducible factor-1 (HIF-1), consisting of the HIF-1α and HIF-1β subunits, is an important transcriptional regulator under hypoxic conditions. It plays an important role in regulating oxygen homeostasis and adaptation to hypoxia in tumor cells. HIF-1α is closely related to aerobic glycolysis in cancer, mainly by regulating HK2 and GLUT1 to participate in cancer cell metabolism and accelerate the Warburg effect [28]. Thus, ectopic expression of miR-210-3p, miR-767-5 and miR-105-5p enhances the Warburg effect in TNBC. miR-210-3p increases HIF-1α activity by targeting glycerol-3-phosphate dehydrogenase 1-like (GPD1L), while targeting cytoglobin (GYGB) to inhibit the activity of the tumor suppressor gene p53 and enhance the Warburg effect [29]. In addition, Jia et al. found that miR-143-5p directly targets HIF-1α-related GLUT1 signaling pathway and that highly expressed miR-143-5p can decrease HIF-1α levels and inhibit the expression of GLUT1 in breast cancer cells, thereby controlling the occurrence of breast cancer [30]. A recent study found that vesicles with low miR-7641 levels derived from cancer-associated fibroblasts (CAFs) also promoted malignant progression of cancer cells by regulating HIF-1α. In contrast, in vesicles with high levels of miR-7641, miR-7641 inhibited HIF-1α to downregulate the levels of HK2, GLUT1, and LDHA to suppress glycolysis in breast cancer [31].

#### Oncogenes

In addition to the HIF-1α pathway, miRNAs can also target oncogenes to regulate glycolysis in breast cancer. The human tumor suppressor gene, p53, plays a key role in regulating glucose metabolism in cells. p53 can inhibit the transition from OXPHOS to glycolysis in cancer cells and block metabolic reprogramming [32]. Studies have shown that Golgi phosphoprotein 3 (GOLPH3L), a highly expressed protein in cancer cells, can stabilize the downstream protein SERPINE1 regulated by p53 to promote glucose metabolism in breast cancer, while miR-1185-2-3p can inhibit the expression of GOLPH3L to prevent the occurrence of breast cancer. Therefore, the discovery of miRNA-GOLPH3L-SERPINE1 pathway provides a new approach for the treatment of breast cancer [33]. Moreover, the downstream regulatory gene NDRG2 of N-myc is a tumor suppressor gene. MiR-181a-5p promotes proliferation and glycolysis of breast cancer cells by downregulating NDRG2 and activating the PTEN/AKT pathway. Silencing of the miR-181a-5p gene effectively reduces the activity of enzymes related to glycolysis, such as HK2, PKM2, and LDHA [34]. In addition, PAICS is considered an oncogene that is also regulated by miRNA. PAICS induces phosphorylation and activation of focal adhesion kinase (FAK). FAK is an activator in breast cancer cells and is related to the glycolytic pathway of breast cancer cells [35]. miR-4731-5p can downregulate PAICS, decrease the levels of PKM2 and GLUT1, and inhibit the phosphorylation of FAK, thereby impeding the aerobic glycolysis of breast cancer cells and inhibiting tumor growth [36].

#### Other molecular mechanisms

Glucose transporters (GLUTs) are important transporters that can mediate glucose transport across the membrane and control the flux of glucose metabolism. They are closely related to glucose metabolism in cancer [37]. GLUTs are usually highly expressed in tumor tissues, resulting in abnormal glucose metabolism in cancer cells. Some miRNAs have been shown to regulate GLUTs to reduce tumor invasion and growth. Genomic analyzes showed that miR-140-5p expression was reduced in breast cancer and directly targeted to decrease GLUT1 levels and exert antitumor effects [38]. miR-Let-7a-5p, a tumor suppressor, inhibits GLUT12-mediated glycolysis in TNBC by targeting its 3′-untranslated region, which includes glucose uptake, lactate and ATP production. The let-7a-5p/GLUT12 axis inhibits the Warburg effect and thus represents a potential therapeutic target for TNBC [39]. In addition, monocarboxylate transporter 1 (MCT1) is upregulated in breast cancer and has a poor prognosis. MCT1 is able to take up lactate and promotes OXPHOS and angiogenesis in tumors. In TNBC, miR-342-3p expression is negatively correlated with MCT1 expression. miR-342-3p is downregulated in TNBC and alters lactate and glucose fluxes in breast cancer by targeting MCT1 upregulation, thereby disrupting metabolic homeostasis. A growing body of research has shown that targeting MCT1 can lead to tumor treatment, and the subtle relationship between miR-342-3p and MCT1 in TNBC also offers a potential treatment strategy for breast cancer [40, 41]. Another notable tumor suppressor miR-101-3p is overexpressed in TNBC and directly targets the protein level of AMP-activated protein kinase (AMPK). Overexpression of AMPK in TNBC may enhance glucose uptake and Warburg effect in cancer cells and promote proliferation of TNBC cells. The miR-101-3p-AMPK axis is a key pathway in the regulation of tumor progression. Overexpression of miR-101-3p and suppression of AMPK protein effectively slow glucose metabolism and inhibit cell proliferation in breast cancer [42] (Table 1).

**Table 1:** Regulation of glycolytic pathways and related molecules by miRNAs in breast cancer cells

miRNA	Target	Glycolysis		Mechanism	Referencess
		Up	Down		
miR-210-3p	HIF-1α/ p53	✓		Increase the activity of HIF-1α and inhibit p53	[29]
miR-143-5p	HIF-1α pathway		✓	Target HIF-1α to inhibit GLUT1 expression	[30]
miR-1185-2-3p	GOLPH3L-SERPINE1		✓	–	[33]
miR-181a-5p	PTEN/AKT pathway	✓		Activate the PTEN/AKT signaling pathway	[34]
miR-4731-5p	PAICS		✓	Inhibit the activity of Focal adhesion kinase	[36]
miR-342-3p	MCT1	✓		Increase the flux of lactate and glucose	[40]
miR-101-3p	AMPK		✓	Downregulates the level of AMPK protein	[42]

## LncRNAs in breast cancer glucose metabolism

LncRNA is an RNA molecule with a length of more than 200 bp and lacks an obvious open reading frame; hence, it does not have the function of the coding protein. Many studies have shown that lncRNAs are closely related to the occurrence of human diseases, and some lncRNAs have been found to regulate the proliferation and migration of cancer cells [43]. In addition, lncRNAs can indirectly participate in a variety of physiological processes by acting as competitive endogenous RNAs (ceRNAs) or “sponges” competitively binding mRNAs, resulting in altered expression levels of downstream target genes. Emerging evidence suggests that lncRNAs may be involved in regulating glucose metabolism in breast cancer cells and play an important role in the development of breast cancer [44].

### LncRNA directly regulates glycolytic enzymes in breast cancer cells

**Fig. 3:**
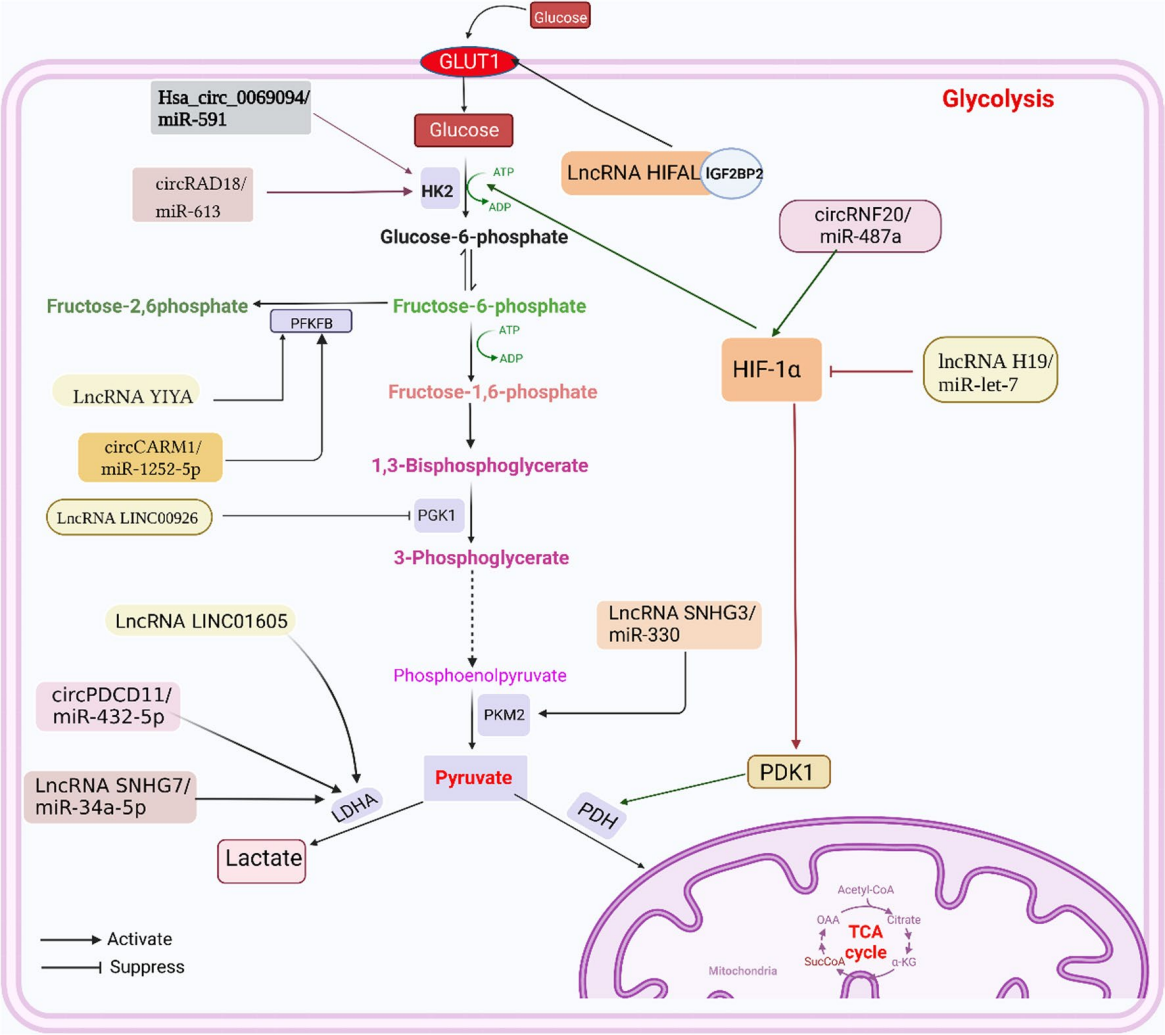
lncRNA and circRNA can regulate glucose metabolism enzymes either directly or by acting as miRNA sponges, thus affecting the glucose metabolism process in breast cancer cells

Similar to miRNAs, lncRNAs can also directly or indirectly regulate the activity of glycolytic enzymes, such as LDHA, PGK1, and PFKFB, in tumor cells. Several studies have shown that lncRNAs LINC01605, LINC00926, LINC00538 (YIYA), and lncRNA Neat1 are closely related to the level of glycolytic enzymes in breast cancer. In recent years, LncRNALINC01605 has been found to be upregulated in a variety of cancers and to accelerate tumor proliferation. Wang et al. found that LINC01605 promoted proliferation and invasion of TNBC cells in vitro and directly targeted the upregulation of LDHA expression [45]. In addition, Chu et al. used relevant breast cancer data to search for lncRNAs that negatively regulate PGK1, and experimentally confirmed that LncRNALINC00926 downregulates breast cancer cell proliferation and metastasis by enhancing E3 ligase STUB1-mediated ubiquitination of PGK1 and downregulating the level of glycolytic reprogramming in cancer cells. In breast cancer, the level of LINC00926 is regulated by the transcription factor FOXO3A, which is able to exert potent tumor suppressive effects. The FOXO3A/LINC00926/PGK1 axis effectively inhibits the Warburg effect in breast cancer and is a potential therapeutic target for breast cancer [46]. The LncRNA YIYA binds to cyclin-dependent kinase 6 (CDK6) and regulates glucose metabolism by regulating CDK6-dependent phosphorylation of PFKFB3. Suppression of YIYA effectively inhibits glucose consumption and lactate production in breast cancer cells [47]. Furthermore, lncRNA Neat1 is a novel cancer-associated lncRNA that is highly expressed in various cancers and plays a major role in breast cancer proliferation, invasion, and metastasis [48]. Park et al. investigated the regulation of breast cancer gluconeogenesis by RNA interference (RNAi) targeting two isoforms of NEAT1 in the highly glycosylated human breast cancer cell line BT -474, and found that Neat1 first binds to PGK1 and upregulates a number of glycolytic enzymes in breast cancer, such as phosphoglyceramylase 1 (PGAM1) and alpha-enolase (ENO1) [49]. In addition, 3-phosphoinositide-dependent kinase 1 (PDK1) is a downstream target of HIF-1α and is upregulated in many cancers. PDK1 can phosphorylate the E1 α-subunit of pyruvate dehydrogenase (PDH) and block the conversion of pyruvate to acetyl coenzyme A, thereby inhibiting the aerobic oxidation of glucose [50]. Peng et al. found that lncRNA H19 acts as a sponge for miR-let-7 to decrease the level of HIF-1α and increase the level of PDK1 in breast cancer cells. This indicates that PDK1 can be used as a target for the treatment of breast cancer [51] (Fig. 3 demonstrates the regulatory role of lncRNA in breast cancer glycolysis).

### LncRNAs regulate gluconeogenic pathways in breast cancer cells

#### HIF-1α signaling pathway

As mentioned above, the HIF-1α pathway plays an indispensable role in glucose metabolism in cancer cells. Some lncRNAs regulate glucose metabolism in breast cancer cells by regulating the HIF-1α pathway. Hypoxia-induced lncRNA MIR210HG promotes the Warburg effect in TNBC by enhancing HIF1α mRNA. Knockdown of MIR210HG also downregulated the expression of proteins, such as GLUT1, PKM2, and LDHA, and the discovery of the HIF-1α/MIR210HG axis may also provide a direction for the treatment of breast cancer [52]. As a novel type of lncRNA, cytoplasmic lncRNA LINK- An is widely distributed in the cytoplasm of various cancers and promotes the reprogramming of TNBC glycolysis by relying on the HIF-1α signaling pathway, highlighting the important role of lncRNA in tumors [53]. Moreover, HIF-1α antisense lncRNA HIFAL can enhance the deactivation of HIF-1α, while HIF-1α can also induce the transcription of HIFAL, forming a positive feedback loop. Targeted inhibition of HIF-1α and HIFAL effectively inhibits cancer [54]. HOXC-AS3 is a carcinogenic lncRNA. By binding to sirtuin6 (SIRT6), it promotes acetylation of H3K9ac, a gene related to energy metabolism and upregulates the levels of PDK4, LDHA, and phosphofructokinase-1 (PFK1) in breast cancer cells, thereby promoting metabolic reprogramming in breast cancer. Moreover, HOXC-AS3 is functionally dependent on HIF-1α, and knockdown of HOXC-AS3 can significantly inhibit the cancer-promoting effect of HIF-1α. Glucose deficiency- induced SP1 can activate the transcription of HOXC-AS3, whereas HOXC-AS3 further acts directly on miR1224-5p. Therefore, HOXC-AS3, miR1224-5p and SP1 form a positive feedback loop, suggesting that HOXC-AS3 plays an important role in the malignant development of breast cancer [55].

#### MYC pathway

The transcription factor MYC is a proto-oncogene that can maintain several processes of cancer metabolism and plays an important role in regulating cancer genes related to glucose metabolism Its family members are mainly divided into C-MYC, N-MYC, and L-MYC [56]. Several lncRNAs have been found to influence cancer progression by interacting with MYC. For example, downregulation of LncRNAFGF13-AS1 has been associated with poor prognosis in breast cancer. FGF13-AS1 has been found to play a predominantly oncogenic role in breast cancer, reducing the glycolytic and stemness properties of breast cancer cells by inhibiting MYC expression through binding to insulin-like growth factor 2 mRNA-binding proteins (IGF2BPs). MYC acts as a negative regulator of lncRNA to inhibit transcription of FGF13-AS1, forming an FGF13-AS1/IGF2BPs/MYC feedback pathway to inhibit breast cancer development and glycolysis [57]. c-MYC regulates gene expression through MAX and is an overactive gene in cancer cells. There is a C-myc binding site in the promoter region of LncRNATSPEAR-AS2, and C-myc can bind to TSPEAR-AS2 and upregulate its expression. TSPEAR-AS2 upregulates GLUT1 and promotes reprogramming of glucose metabolism in breast cancer by binding to IGF2BP2, leading to poor prognosis. Knockdown of TSPEAR-AS2 effectively inhibits breast cancer cell growth and aerobic glycolysis, suggesting that targeting the C-myc/TSPEAR-AS2/GLUT1 axis has important potential research value for the treatment of breast cancer [58, 59].

#### Hippo/YAP signaling pathway

The Hippo signaling pathway is primarily involved in the regulation of cell proliferation, survival, and migration, and its dysregulation has beenassociated with abnormal cell growth and tumorigenesis. YAP/TAZ is the main effector of the Hippo pathway, and Hippo/YAP signaling can regulate a variety of cellular metabolic pathways, including glucose, lipid, nucleotide, and amino acid metabolism [60]. A growing number of studies have demonstrated the important role of the Hippo/YAP pathway in cancer development. Studies have shown that YAP-targeted upregulation of lncBCAR4 promotes reprogramming of glucose metabolism in breast cancer cells. BCAR4 acts together with the Hedgehog pathway effector GLI2 to upregulate HK2 and PFKFB3, which promotes proliferation and glucose uptake in breast cancer cells [61]. Moreover, LncRNAGHET1 was induced under hypoxic conditions, leading to excessive activation of the Hippo/ YAP pathway, directly accelerating TNBC cell proliferation and glycolysis [62]. In recent years, he Hippo/ YAP signaling network has been continuously improved and explored, and its role in cancer has gradually become a research focus. Targeting the Hippo/ YAP signaling pathway is considered as a potential strategy for cancer treatment (Table 2).

**Table 2:** Regulation of lncRNAs in the glycolytic pathway and related molecules in breast cancer cells

lncRNA	Target	Glycolysis		Mechanism	References
		Up	Down		
LncRNA MIR210HG	HIF-1α pathway	✓		Upregulates GLUT1, PKM2, and LDHA	[52]
LncRNA LINK-A	HIF-1α pathway	✓		–	[53]
LncRNA HOXC-AS3	HIF-1α pathway	✓		–	[55]
LncRNA FGF13-AS1	IGF2BPs/Myc axis		✓	Inhibit the expression of Myc	[57]
LncRNA TSPEAR-AS2	C-myc	✓		Upregulates GLUT1	[58]
LncRNA BCAR4	Hippo and Hedgehog pathway	✓		Upregulates HK2 and PFKFB3	[61]
LncRNA GHET1	Hippo/YAP signaling pathway	✓		–	[62]

### LncRNA regulates breast cancer glucose metabolism via ceRNA

lncRNAs can also function as ''miRNA sponges.'' Through the miRNA response element, it competitively binds miRNA with the target miRNA through complementary base pairing, reducing the level of free miRNA and weakening the "silencing effect" of miRNA on its target mRNA, thus realizing the regulation of the target mRNA [63]. In breast cancer, some lncRNAs downregulate miRNA levels by exerting oncogenic effects, thereby affecting cancer cell invasion, proliferation and metastasis. It was found that lncRNA SNHG3, lncRNA SNHG5, and lncRNA SNHG7 regulate glucose metabolism in breast cancer cells by acting as miRNA sponges. LncRNASNHG3 is an exosome secreted by CAFs that inhibits mitochondrial activity in breast cancer cells, promoting glycolysis in cancer cells. SNHG3 acts as a sponge that inhibits miR-330 expression and increases PKM protein levels in cancer cells. The SNHG3/miR330 axis mainly regulates PKM at the post-transcriptional level to promote glycolysis and proliferation in breast cancer [64]. Similarly, lncRNA SNHG5 directly targetsmiR-299 repression, upregulates BTB domain and CNC homology 1 (BACH1) transcription factor levels, and increases HK2 and PFK1 [65], and lncRNA SNHG7 directly targets miR34a-5p to upregulate LDHA and promote glycolysis in breast cancer [66]. In addition, lncRNA LINC00346was found to be closely related to GLUT1, and knockdown of LINC00346 reduced glucose uptake in breast cancer cells and slowed breast cancer malignancy by directly targeting miR-148a/b to inhibit GLUT1 [67]. Transcription factor-activating enhancer binding protein 2 alpha (TFAP2A) is upregulated as an oncogene in many cancers, including breast cancer. Using tissues and cells from patients with breast cancer, Ding et al. found that TFAP2A could be regulated by the lncRNA MAFG-AS1/miR-3196 axis. The lncRNA MAFG-AS1 acts as a sponge for miR-3169 and TFAP2A is a target of miR-3169, and knocking down MAFG-AS1 could lead to inactivation of the JAK2/ STAT pathway, thereby inhibiting breast cancer progression [68].

Some lncRNAs may not only act as oncogenes and promote tumor progression but also have tumor suppressive functions. LncRNA Parentally Expressed Gene 3 (MEG3) isa transcription factor RNA that is expressed in a variety of normal tissues but whose expression is absent or downregulated in human tumors. In recent years, MEG3 has been found to interact with miRNAs at sufficient levels to affect cell proliferation, apoptosis, and angiogenesis [69]. In breast cancer, MEG3 acts as a tumor suppressor and directly inhibits miR-21 expression via the PI3K/Akt pathway, slowing the Warburg effect in tumors [70]. Jin et al. found that low expression of MBNL1-AS1 in breast cancer predicted poor prognosis of breast cancer. MBNL1-AS1 significantly decreased the expression of miR-889-3p, while KLF9, a highly conserved zinc finger protein with a tumor suppressive effect, was a downstream target of miR-889-3p and negatively regulated by miR-889-3p. As miR-889-3p decreased, its inhibitory effect on KLF9 was attenuated. This indicates that the MBNL1-AS1/miR-889-3p/KLF9 axis effectively reduces glycolysis in breast cancer cells and inhibits breast cancer progression [71] (Table 3).

**Table 3:** LncRNA regulates breast cancer glucose metabolism via ceRNA

LncRNA	Target miRNA	Target of miRNA	Function	References
LncRNA SNHG3	miR-330	PKM	Up	[64]
LncRNA SNHG5	miR-299	BACH1	Up	[65]
LncRNA SNHG7	miR-34a-5p	LDHA	Up	[66]
LncRNA LINC00346	miR-148a/b	GLUT1	Down	[67]
LncRNA MAFG-AS1	miR-3196	TFAP2A	Up	[68]
LncRNA MEG3	miR-21	–	Up	[70]
lncRNA MBNL1-AS1	miR-889-3p	KLF9	Down	[71]

## CircRNAs in breast cancer glucose metabolism

CircRNA is a novel ncRNA with a covalent closed-loop structure lacking 5' and 3' ends and is not affected by RNA exonucleases; therefore, its expression is stable [72]. CircRNAs are abnormally expressed in tumors, are important regulators of tumor progression, and have been shown to regulate the Warburg effect in cancer cells [73]. The current regulatory mechanisms of circRNAs can be divided into four main areas: They act as molecular miRNA sponges, regulate gene transcription, regulate selective RNA shearing, and interact with proteins. In breast cancer, circRNAs mainly act as miRNA "sponges" and ceRNAs compete with miRNAs to regulate proliferation, invasion and glycolysis of breast cancer cells [74].

### CircRNA as a miRNA sponge for the regulation of glycolytic enzymes

As a key enzyme in glucose metabolism, HK2 is an important molecule in glycolysis. In recent years, a number of circRNAs have been shown to act as miRNA sponges in HK2 under pathological conditions in breast cancer, such as circRNF20, circRAD18 and Hsa_circ_0069094 [75–77]. Among them, circRNF20 binds to miR-487a and promotes the expression of HIF-1α, thereby upregulating HK2 levels and accelerating the Warburg effect in breast cancer cells. circRAD18 functions as a miR-613 sponge to upregulates HK2 and further exacerbate breast cancer. Hsa_circ_0069094 is upregulated in breast cancer and regulates HK2 expression by interacting with miR-591. It has been reported that Hsa_circ_0069094 can also upregulate the expression of The high mobility group A1 (HMGA1) by negatively regulating miR-661, which accelerates glycolysis in breast cancer cells [78]. HMGA1 is a non-histone chromatin structural protein that predicts poor prognosis in malignant tumors [79]. In addition, circRNAs can regulate LDHA expression in breast cancer cells. In a study on TNBC, Xing collected tissues from TNBC patients for examination and found that circPDCD11, a sponge of miR-432-5p, upregulated LDHA in triple negative breast cancer, and further experiments showed that knockdown of circPDCD11 effectively inhibited tumor growth [80]. In another study, circYY1 was found to upregulate the protein levels of HK2 and LDHA simultaneously in breast cancer cells and knockdown of circYY1 has a good tumor inhibitory effect [81]. Therefore, targeting circYY1 may be a potential strategy for the treatment of breast cancer As an enzyme that regulates glycolysis, PFKFB may also be regulated by circRNA. CircCARM1 is secreted by tumor stem cells (Cancer Stem Cells, CSC). Studies have shown that circCARM1, derived from BCSC exosomes, can upregulate PFKFB2 expression and promote glycolysis in breast adenocarcinomas by competitively binding to miR-1252-5p. miR-1252-5p has the function of targeting PFKFB2 for degradation, but is inhibited by circCARM1 [82]. (Fig. 3 demonstrates the regulatory role of circRNA in breast cancer glycolysis).

### CircRNA as a miRNA sponge for the regulation of other molecules

In breast cancer, the level of HIF-1α is closely associated with circRNAs. CircRBM33 and circZFR, as sponges of miR-542-3p, upregulate HIF-1α, accelerate the glycolysis of breast cancer cells, and promote the proliferation and metastasis of breast cancer cells [83, 84]. Moreover, circRNF20 has been shown to affect the level of HIF-1α and exacerbate the malignancy of breast cancer [75]. Blocking or inhibiting HIF-1α is considered an effective way to treat tumors, and a considerable number of HIF-1α inhibitors have been used as anticancer drugs and in clinics.

In addition, studies have shown that circRNAs can also regulate related proteins, genes, and transcription factors through the "sponge effect" of miRNAs to affect glucose metabolism in tumor cells. Spindle and kinetochore-associated protein 2 (SKA2) is a cell cycle regulatory protein that is upregulated in a variety of malignant tumors and is considered a tumor promoter. SKA2 has been associated with breast cancer metastasis [85]. A study by Dou et al. suggested that circ_0008039 negatively regulates miR-140-3p, thereby upregulating SKA2 expression and accelerating aerobic glycolysis in breast cancer cells. Therefore, circ_0008039 is considered an oncogene that promotes tumor progression [86].

Circ_0072995 is a new circRNA discovered in recent years. Using tumor tissues and cells from breast cancer patients, Qi et al. found that circ_0072995 binds competitively to miR-149-5p to promote serine hydroxymethyl transferase 2 (SHMT2) expression and accelerate glucose uptake in cancer cells. miR-149-5p can directly target SHMT2 mRNA in breast cancer cells. SHMT2 is highly expressed in many tumors and is considered a potential new target for tumor therapy [87]. In addition, GLUT1, which plays an important role in reprogramming glucose metabolism in tumor cells, can also be regulated by circRNAs. In breast cancer, circ_0001955 acts as a sponge for miR-1299. miR-1299 was able to inhibit GLUT1 expression, but miR-1299 levels were downregulated by circ_0001955, resulting in increased GLUT1 levels [88].The transcription factor ALX4 is a tumor suppressor that plays an important inhibitory role in breast cancer development. CircKLHL24 can inhibit the proliferation and migration of breast cancer cells, downregulate the levels of HK2, GLUT1 and LDHA, and upregulate the expression of ALX4 by competitively binding miR-1204, thereby achieving tumor inhibition [89]. In general, with the continuous progress of research, an increasing number of genes and molecules are regulated by circRNAs, providing new ideas and clues for cancer treatment (Table 4).

**Table 4:** circRNA regulates glycolytic enzymes and pathways in breast cancer cells by acting as “miRNA sponges”

CircRNA	Target miRNA	Target of miRNA	Function	References
circRNF20	miR-487a	HIF-1α	Up	[75]
circRAD18	miR-613	HK2	Up	[76]
Hsa_circ_0069094	miR-591	HK2	Up	[77]
Hsa_circ_0069094	miR-661	HMGA1	Up	[78]
circPDCD11	miR-432-5p	LDHA	Up	[80]
circCARM1	miR-1252-5p	PFKFB2	Up	[82]
circRBM33	miR-542-3p	HIF-1α	Up	[83]
circZFR	miR-578	HIF-1α	Up	[84]
Circ_0008039	miR-140-3p	SKA2	Up	[86]
Circ_0072995	miR-149-5p	SHMT2	Up	[87]
circ_0001955	miR-1299	GLUT1	Up	[88]
circKLHL24	miR-1204	ALX4	Down	[89]

## Clinical implications of targeting glucose metabolism in BC

The use of metabolic regulation in breast cancer in clinical treatment seems promising. Several inhibitors of aerobic glycolysis of cancer cells have recently been used clinically and contributed to improved outcomes in drug-resistant cancer therapy. Some ncRNAs involved in the regulation of glycolysis in breast cancer cells have emerged as potential therapeutic targets and prognostic biomarkers for breast cancer. For example, miR-125a-5p, miR-145, and miR-34a have been shown to be effective in the treatment of breast cancer, and miR-30c and miR-339-5p play important roles in predicting treatment outcome in breast cancer [90, 91]. In addition, Zou et al. identified glycolysis-related lncRNAs using bioinformatics and other methods and constructed a prognostic model based on prognostic genes for breast cancer. They showed that glycolysis-related lncRNAs play an important role in facilitating individualized survival prediction in patients with breast cancer, which is a new potential prospect for breast cancer [92].

Recently, it was discovered that non-coding RNAs successfully control tumor drug resistance and also serve as prognostic markers to aid diagnosis. Aerobic glycolysis in cancer cells has been linked to drug resistance, and combining multidrug resistance of tumors with glycolysis of tumor cells to combat tumors is a new line of thought [93]. The lncRNA ANRIL is associated with a number of malignancies and is a useful prognostic factor. Jianli et al. discovered that lncRNA ANRIL was significantly upregulated in TNBC patients, possibly enhancing glycolysis and doxorubicin resistance. When ANRIL is knocked down, TNBC cell glycolytic activity and drug resistance can be successfully reduced, which is crucial for the treatment of TNBC patients and represents a new target for the drug treatment of breast cancer tumors [94]. Tamoxifen resistance has been linked to increased glycolysis in cancer cells in research on ER -positive breast cancer. Tamoxifen increases EREG gene expression by inhibiting miR-186-3p, which is essential for tamoxifen-resistant breast cancer cells to induce glycolysis. In this study, it was found that systemic administration of Agomir-186-3p could significantly reduce glycolysis and drug resistance in breast cancer cells, and that miR-186-3p and EREG were negatively correlated in human breast cancer samples, demonstrating the clinical correlation of the miR-186-3p/EREG axis in human breast cancer. It has been suggested that miR-186-3p may be a useful target for the treatment of drug resistance in breast cancer [95]. In addition, CircRNF111 may be an important target for the treatment of paclitaxel resistance in breast cancer. Zang et al. discovered that CircRNF111 is overexpressed in paclitaxel-resistant breast cancer cells and functions as a miRNA sponge to negatively regulate miR-140-5p and upregulate E2F transcription factor 3 (E2F3), resulting in paclitaxel-resistant breast cancer cells. Silencing CircRNF111 efficiently restores this process and reduces paclitaxel resistance in breast cancer cells. Finally, non-coding RNAs directly affect glucose metabolism in cancer cells and control drug resistance in cancer cells, indicating a potential clinical treatment target [96]. Non-coding RNAs are increasingly becoming a rising star.

## Conclusions and perspectives

Recently, a growing number of ncRNAs have been found to effectively regulate the glucose metabolism pathway in breast cancer cells. As important regulatory factors in cancer, miRNAs are mainly involved in regulating gene expression and directly or indirectly regulate enzyme systems and pathways of glucose metabolism. With the progress of research, miRNAs have gradually emerged as important targets for cancer therapy. As one of the hotspots of biological research in recent years, lncRNAs can not only regulate enzymes and pathways related to glucose metabolism, but also inhibit the expression of miRNAs as ceRNAs and play a role in promoting or inhibiting cancer. CircRNA is considered a specific ncRNA molecule closely associated with the occurrence and development of a variety of cancers. Exon circRNAs mainly function as miRNA sponges to affect genes related to glucose metabolism in breast cancer, thereby altering the metabolic state of cancer cells.

However, although ncRNA targeting glucose metabolism in breast cancer cells has made tremendous progress at this stage, there are still associated problems in the research process: most ncRNAs function primarily as diagnostic and prognostic markers, and ncRNA therapies targeting glucose metabolism in cancer cells are challenging and not ideal for clinical trials. Currently, about 11 RNA-based therapies are approved, mainly for liver, muscle, and other diseases. Most RNA therapies are in the early clinical phase. Although some progress has been made in the research of miRNAs, the mechanism of some lncRNAs and circRNAs has not been fully elucidated and needs further research. In summary, the transfer of ncRNA molecules to the target is urgently needed in clinical applications. Oligonucleotides are negatively charged and hydrophilic, so they cannot enter the cell membrane by diffusion. Therefore, it is important to develop efficient ncRNA delivery systems. In conclusion, with the continuous development of ncRNA therapy and intervention in cancer cell glycolysis for the treatment of breast cancer, the organic combination of ncRNA and cancer cell glycolysis will provide a new perspective for the treatment of breast cancer. We need to further explore the mechanism by which ncRNAs regulate glucose metabolism in breast cancer cells to find new therapeutic targets and provide a molecular basis and new direction for the precise treatment of breast cancer.

## Declarations

### Data availiability

Data available within the ariticle or its supplementary materials.
